# Nerve transfer to musculocutaneous for elbow flexion restoration in brachial plexus injury (Ulnar and/or Median vs. Intercostal): A systematic review and meta-analysis of comparative studies

**DOI:** 10.1007/s00701-025-06650-0

**Published:** 2025-08-26

**Authors:** Mohamed K. A. Genedy, Esraa Y. Salama, Mohamed Ashraf Elsaadany, Mohamed A. F. AbdelWahab, Ahmed Fathy Amin, Ahmed A. Lashin, Ahmed O. Sabry

**Affiliations:** 1 Faculty of Medicine, Cairo University, Cairo, Egypt; 2 Faculty of Medicine, Benha University, Benha, Egypt; 3 Orthopedic Surgery Department, Cairo University, Cairo, Egypt

**Keywords:** Brachial Plexus Injury, Nerve Transfer, Oberlin, Intercostal, Elbow

## Abstract

**Background:**

Nerve transfers are a cornerstone in the surgical management of traumatic brachial plexus injuries (BPIs) to restore elbow flexion. Common donor nerves include intraplexal sources like the ulnar and median nerves (fascicular transfers) and extraplexal sources like the intercostal nerves (ICNs). Despite the widespread use of both techniques, the optimal donor nerve remains a subject of debate. This systematic review and meta-analysis aims to compare these techniques for restoring elbow flexion in BPIs.

**Methods:**

A systematic search was conducted across PubMed, Embase, Cochrane Library, Scopus, and Web of Science to identify comparative studies. The quality of the studies included was assessed using the Newcastle–Ottawa Scale (NOS). Meta-analyses were performed to compare motor recovery (≥ M3), time to M3 recovery, and complication rates between the two surgical approaches.

**Results:**

The analysis included 13 studies with a total of 537 patients. In the overall cohort, which included mixed injury patterns, fascicular transfers showed a statistically significant advantage for achieving ≥ M3 recovery (RR = 0.84, 95% CI [0.75, 0.94]). However, when the analysis was restricted to patients with only upper-BPIs, there was no significant difference in achieving ≥ M3 strength between fascicular and ICN transfers (RR = 0.92, 95% CI [0.82, 1.04]). Fascicular transfers resulted in a significantly faster time to ≥ M3 recovery by approximately five months (MD = 5.25, 95% CI [2.87, 7.62]). Donor-site morbidity (18 sensory, 10 motor deficits) and wrist co-flexion were reported in fascicular transfer groups, whereas pneumothorax (4 cases) was the primary complication for ICN transfers.

**Conclusion:**

In patients with upper-BPIs, fascicular and ICN transfers yield comparable elbow flexion strength. The choice of procedure is a trade-off between the faster recovery offered by fascicular transfers and the better rehabilitation course of ICN transfers.

**Supplementary Information:**

The online version contains supplementary material available at 10.1007/s00701-025-06650-0.

## Introduction

Brachial plexus injuries (BPIs) present a significant reconstructive challenge, often with devastating physical and socioeconomic impacts [3]. While the incidence of BPI has increased over the past 50 years, often linked to high-energy trauma, microsurgical developments over the last three decades have introduced new modalities to improve clinical outcomes [28]. Traumatic BPI affects about 3% of adult patients admitted to general hospitals and can result in significant functional impairment [11]. Among the various reconstructive goals, the restoration of elbow flexion is consistently a primary objective in the surgical treatment of traumatic BPI in adults, given its importance for daily activities [32]. Nerve transfer, also known as neurotization, was first introduced 70 years ago and has evolved to be one of the most used modalities in BPIs [14].

The donor nerve can be intraplexal such as the cases involving ulnar nerve (UN) usage or it can be extraplexal which includes the usage of the intercosal nerves (ICNs) [27]. Traditionally, reinnervation of the musculocutaneous nerve (MCN) was pursued via nerve grafting, employing donor roots such as C5 or C6 [25]. However, this approach often necessitated lengthy grafts, with suboptimal outcomes in cases involving root avulsions or dense scarring. In such circumstances, surgeons found fascicular transfers to be effective for these cases, as it allowed for expanding the time window for surgery, earlier recovery, and improved outcomes [2].

In the early 1960 s, Seddon and Yeoman first described the ICN-MCN transfer, occasionally utilizing sural nerve interposition graft. In 1968, Tysuyama and Hara modified this technique by directly connecting ICNs to the MCN without interposed grafts, reporting excellent functional outcomes [19].

This direct ICN-MCN transfer became widely adopted for several decades until 1994, when Oberlin first reported the transfer of one or two fascicles from the UN to the biceps branch of the MCN, which has gained rapid acceptance due to its simplicity, proximity of coaptation, and rapid return of elbow flexion [1]. Building on this, Mackinnon later introduced the double fascicular transfer (Oberlin II), incorporating an additional transfer from the median nerve (MN) to the brachialis branch of the MCN [1].

While Oberlin I and II procedures are widely used in the management of BPIs, ICN-MCN transfer remains essential [27]. Owing to their extraplexal origin, they are utilized when nerves within the plexus are already utilized or unavailable, and compromised, which occurs in pan BPIs. ICN-MCN may also be utilized in upper BPI, despite the feasibility of Oberlin I and II, due to donor site morbidity that does not compromise ipsilateral hand function [27]. Although there are many nerve transfer techniques, the optimal method is still controversial, particularly regarding the trade-off between the functional outcome and associated morbidity. Hence, the aim of this systematic review and meta-analysis is to determine the optimal donor nerve in each clinical context.

## Methodology

### Protocol registration

This systematic review and meta-analysis was conducted according to the PRISMA 2020 guidelines [24]. The study followed the guidance of the Cochrane Handbook of Systematic Reviews of Interventions [6]. The protocol was registered in PROSPERO (CRD420251074220).

### Search strategy

On June 11, 2025, a systematic search was performed across PubMed, Embase, Scopus, and Web of Science. The strategy combined terms for "nerve" and "transfer" with specific donor nerves like "median," "ulnar," "Oberlin," and "intercostal." As present in the table (Online Resource 1).

### Eligibility criteria

Studies included focused on traumatic BPIs in adults with impaired elbow flexion and directly comparing the postoperative outcomes of UN-MCN and/or MN-MCN with ICN-MCN transfers for elbow flexion restoration. Exclusions were conference papers, secondary studies, non-interventional studies, studies employing interposition grafts, birth injuries, single-arm studies, and non-nerve or irrelevant nerve transfer studies.

### Study selection

A systematic search identified articles, with duplicates removed manually on Rayyan web app [26]. Two independent, blind reviewers screened titles/abstracts, then full texts, using Rayyan. Discrepancies were resolved by discussion or a third reviewer.

### Data extraction

Data extraction was conducted independently by two authors using standardized spreadsheets, capturing demographic information and both primary and secondary outcomes. Key outcomes included Medical Research Council (MRC) grades (≥ M3), time to M3 recovery, and muscle reinnervation (reported as mean ± standard deviation (SD)). Complications such as pneumothorax, wrist-elbow co-contraction, and donor morbidities were also recorded, noting either the total number of events or proportions. Any discrepancies between the independent extractions were resolved through discussion and consensus between the authors.

Complications that were not mentioned in a study were noted as "NR" (not reported), while those mentioned without specified proportions were designated "NS" (not specified). For continuous data, if presented as a mean and range, conversion to mean ± SD was performed using Walter and Yao’s [33] formula, and if presented as median and range Wan’s [34] formula was employed.

### Quality assessment

Two authors assessed the quality independently; any disagreements were resolved by discussion or by a third experienced author. The NOS tool [23] contains three main domains: selection of the participants, comparability between groups and exposure, with a total of nine items. Each answer has a judgment of “yes” or "no." A score between 1 and 9 is given to each study.

The certainty of evidence retrieved from our meta-analysis will be assessed using the GRADE (Grading of Recommendations, Assessment, Development, and Evaluation) protocol [10]. This approach evaluates the evidence based on, risk of bias, inconsistency, indirectness, imprecision, and publication bias. The overall certainty of evidence for each outcome will then be classified as very low, low, moderate, or high, providing a transparent measure of confidence in our findings.

### Statistical analysis

Statistically analyses were performed in R [22], using meta [29] and metafor [13] packages. Continuous outcomes were analyzed using mean difference (MD) and dichotomous outcomes using Risk Ratios (RR), both with 95% confidence intervals (CIs). For dichotomous outcomes, the proportion of patients achieving functional MRC grades (≥ M3), and risk ratios (RR) with a 95% CI will be utilized. Statistical heterogeneity between studies and subgroups will be evaluated using the I^2^ statistic and Cochran's Q test (Chi^2^ test's *p*-value). I^2^ values of < 50%, and > 50% will be categorized as low, and high heterogeneity, respectively. A p-value of ≤ 0.05 will imply statistically significant effect size and a p-value of ≤ 0.05 imply non-significant heterogeneity. Random effect model and leave-one-out were employed only when heterogeneity was significantly high. Publication bias was visually assessed using funnel plots for outcomes with enough included studies (*n* ≥ 10).

## Results

### Search results and study selection

**Fig. 1:**
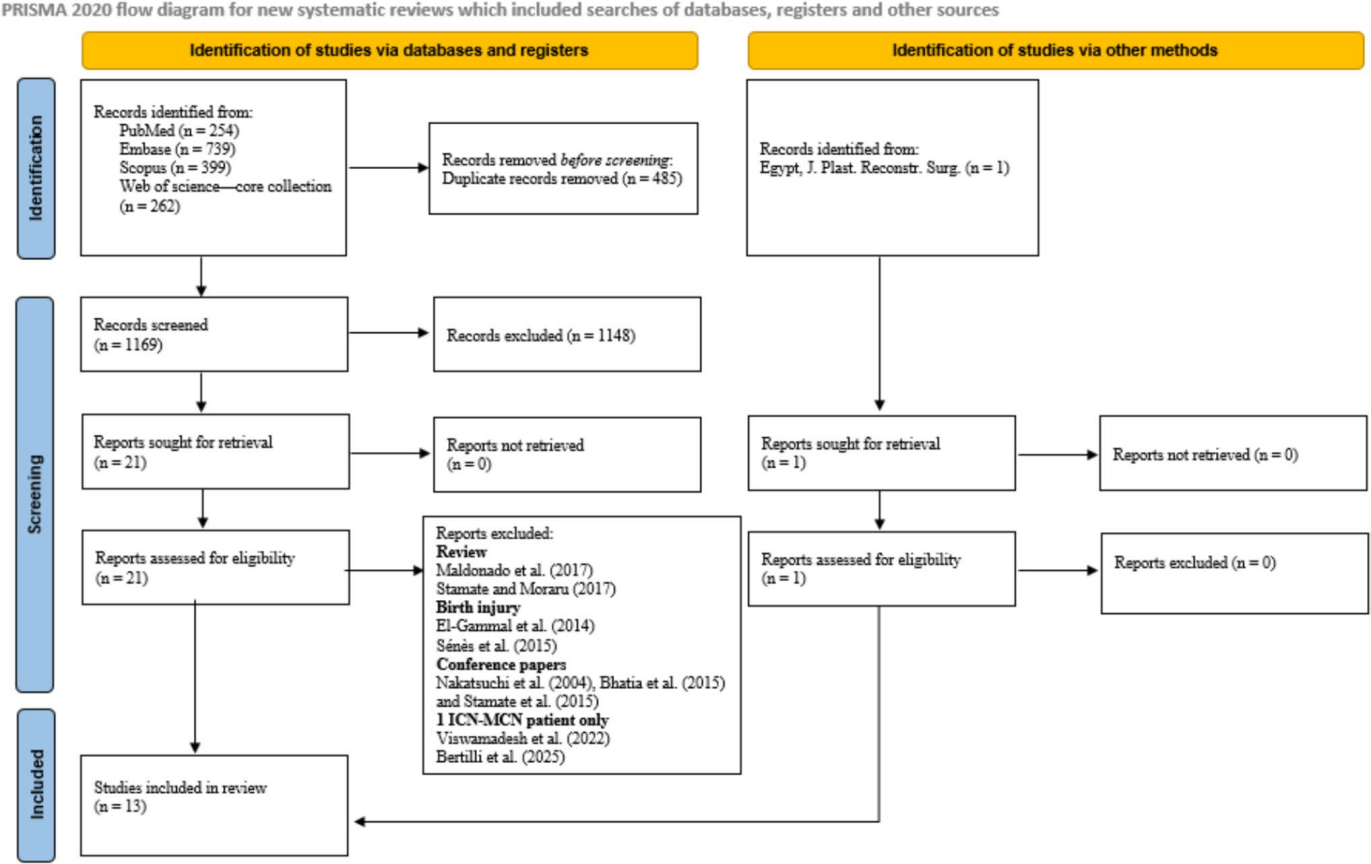
PRISMA flow chart

Four databases were searched, which identified 1654 records. After 485 duplicates were removed, 1169 records were left for screening. Of these, 1148 were excluded based on title and abstract. The full texts of the remaining 21 reports, plus one additional report identified manually, were assessed for eligibility. From this, nine reports were excluded for reasons such as being a review, a conference paper, or having a birth BPIs population. Ultimately, 13 studies [4, 5, 7, 9, 12, 15, 16, 18, 20, 21, 27, 30, 31] were included in the final review (Fig. 1).

### Characteristics of included studies

Table (Online Resource 2) presents a summary of the 13 included studies [4, 5, 7, 9, 12, 15, 16, 18, 20, 21, 27, 30, 31] in this review, involving a total of 537 patients. These studies were conducted across a range of countries, including Thailand, India, Japan, France, Argentina, Iran, Egypt, Singapore, Taiwan, and Romania, with publication dates spanning from 2005 to 2025. UN and/or MN transfers, mostly in the form of Oberlin I and II procedures, were compared against ICN transfers. Most of the studies were retrospective, with two being prospective cohorts. Sample sizes for the intervention groups varied widely, from as few as 3 to as many as 68 patients. Where reported, the mean time to surgery ranged from approximately 1.5 to 6.7 months, while follow-up periods spanned from 13 to over 60 months.

The patient cohorts primarily consisted of individuals with upper or upper extended (*n* = 430) BPIs, with Pan-BPIs being less frequent (*n* = 107). The mean age of patients, where available, generally ranged from the mid-20 s to late-30 s, and across the studies that provided gender data, there was a notable predominance of male participants (256 males to 30 females) (Online Resource 3).

### Quality assessment

A methodological quality assessment of the 13 included studies [4, 5, 7, 9, 12, 15, 16, 18, 20, 21, 27, 30, 31] using the Newcastle–Ottawa Scale (NOS) reveals a collection of evidence that is of predominantly moderate quality with a range from poor to good. Generally, the research performed well across domains, however, a significant methodological flaw was prevalent. Six studies [9, 12, 20, 21, 30, 31] appeared to use selective intervention, creating flawed comparison, where ICN-MCN employed for pan-BPIs while fascicular transfers to patients with less upper-BPIs, without adjusting for this fundamental difference (Online Resource 3 and Online Resource 4). Certainty of evidence is demonstrated in a GRADE evidence profile (Online Resource 5).

### Meta-analysis results: Overall studies

**Fig. 2:**
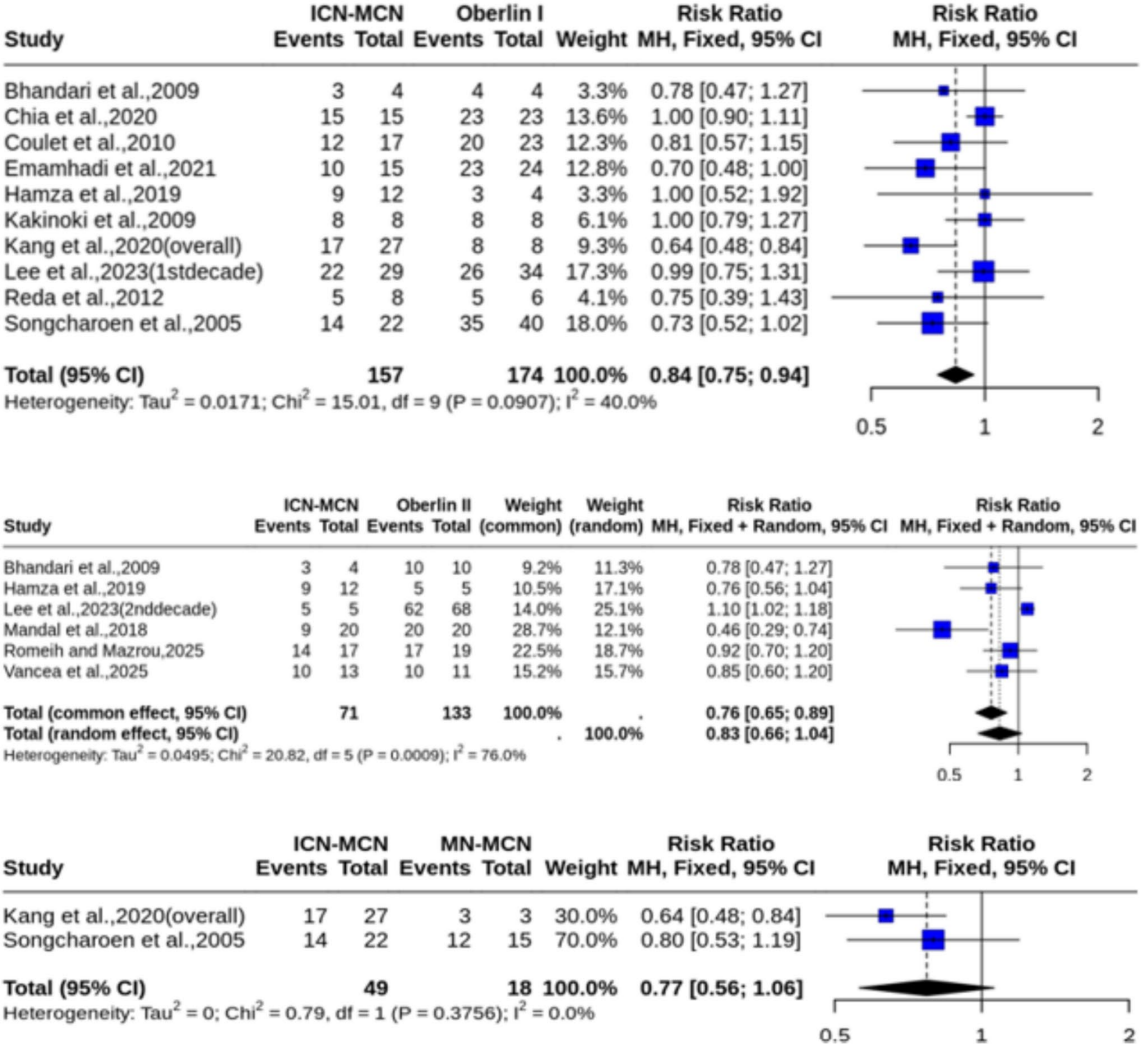
Forest plots of motor recovery outcomes in the overall cohort. **A** Risk ratio for achieving ≥ M3 recovery (Oberlin I vs. ICN-MCN). **B** Risk ratio for achieving ≥ M3 recovery (Oberlin II vs. ICN-MCN). **C** Risk ratio for achieving ≥ M3 recovery (MN-MCN vs. ICN-MCN)

When analyzing the overall studies [4, 5, 7, 9, 12, 15, 16, 18, 20, 21, 27, 30, 31], which included both pan-BPI and upper-BPI cases, the Oberlin I technique demonstrated significant effects. A meta-analysis of ten studies [4, 5, 7, 9, 12, 15, 16, 18, 20, 30] (*n* = 331) for achieving ≥ M3 motor grade showed a significant effect favoring the Oberlin I procedure (RR = 0.84, 95% CI [0.75, 0.94]), with no significant heterogeneity (I^2^ = 40.0%, *p* = 0.09) (Fig. 2A), likely due to selection bias present in some of the studies included in this comparison. Visual inspection of the funnel plot revealed a slight skew toward smaller studies reporting larger effects, and Egger’s test did not reach statistical significance (p = 0.06). While this finding does not confirm publication bias, the borderline result suggests that small‑study effects cannot be entirely excluded (Online Resource 6).

Analyses for the Oberlin II technique showed no statistically significant difference for achieving ≥ M3 motor recovery (RR = 0.83, 95% CI [0.66, 1.04]), with both outcomes marked by substantial heterogeneity (I^2^ = 76.0% and I^2^ = 64.5%, respectively) (Fig. 2B). A sensitivity analysis, however, revealed the study by Lee et al. (2023) [18] to be a significant outlier. Once this study was omitted, the result for the ≥ M3 outcome became statistically significant (RR = 0.77, 95% CI [0.63, 0.94]), with heterogeneity was reduced to I^2^ = 38.3% (Online Resource 7). The comparison between MN and ICN transfers for achieving ≥ M3 (2 studies, *n* = 67) was also not significant (RR = 0.77, 95% CI [0.56, 1.06]) and showed no heterogeneity (I^2^ = 0%) (Fig. 2C).

### Meta-analysis results: Upper-BPI studies and subgroups

**Fig. 3:**
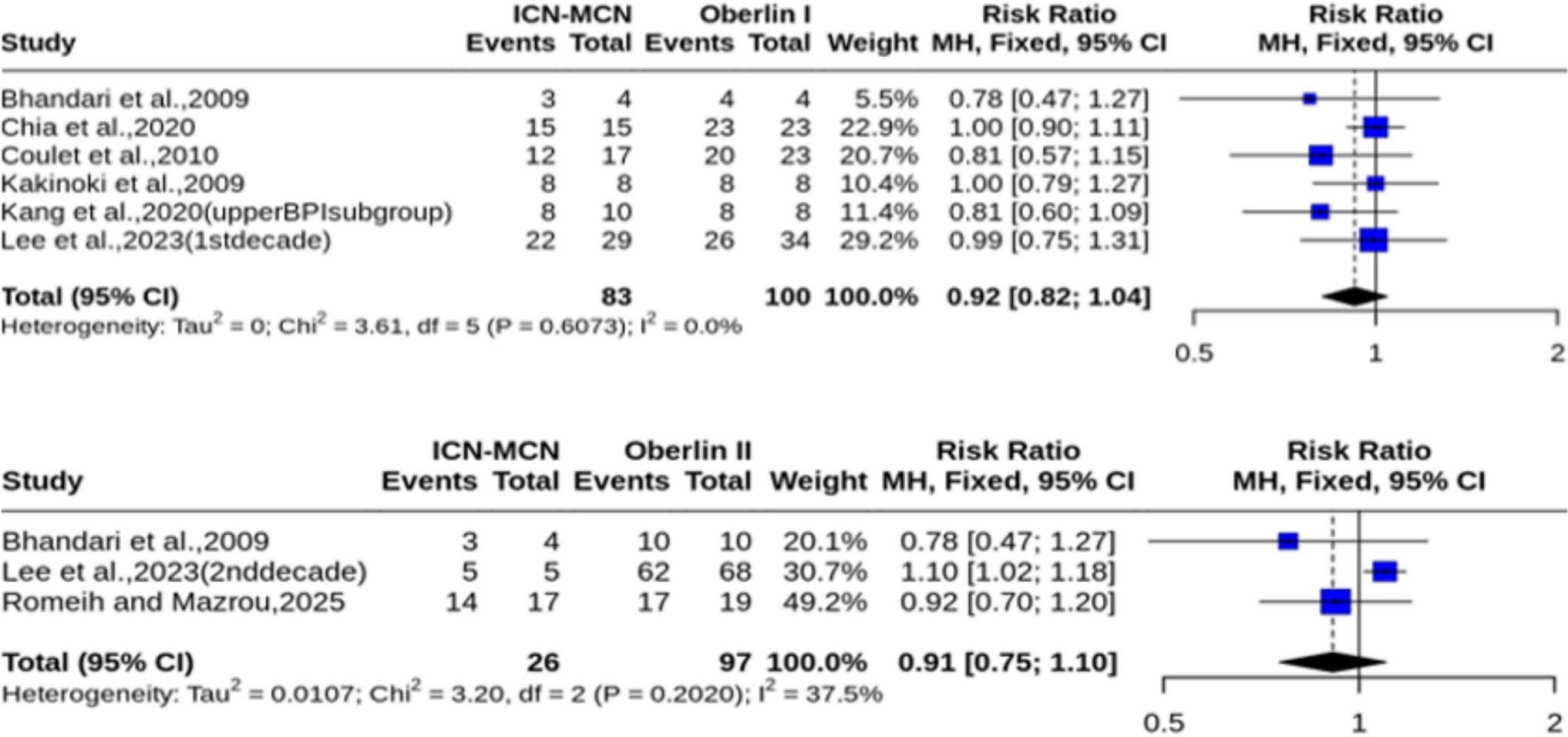
Forest plots of motor recovery outcomes in the upper-BPI cohort. **A** Risk ratio for achieving ≥ M3 recovery (Oberlin I vs. ICN-MCN). **B** Risk ratio for achieving ≥ M3 recovery (Oberlin II vs. ICN-MCN)

When the analysis was restricted to upper-BPI studies and subgroups [4, 5, 7, 15, 16, 18, 27]. the Oberlin I technique, neither the analysis for achieving ≥ M3 (6 studies, *n* = 183; RR = 0.92, 95% CI [0.82, 1.04]) with no heterogeneity (I^2^ = 0%) (Fig. 3A). Similarly, for the Oberlin II technique in upper-BPI studies, the analysis for achieving ≥ M3 did not reach statistical significance (3 studies, *n* = 123; RR = 0.91, 95% CI [0.75, 1.10]), nor significant heterogeneity (I^2^ = 37.5%, *p* = 0.2) (Fig. 3B).

**Fig. 4:**
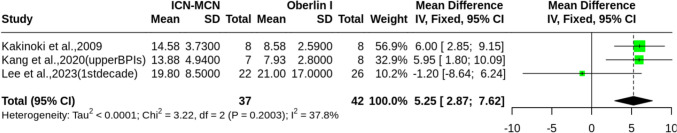
Forest plot of mean difference in time to achieve ≥ M3 in the upper-BPI cohort (Oberlin I vs. ICN-MCN). Note: 1 patient lost follow up in the middle of the Kang study, this patient was excluded from this analysis

Time to reinnervation and recovery outcomes were exclusively reported in upper-BPI studies. For the time to achieve ≥ M3, a meta-analysis of three studies [15, 16, 18] (*n* = 79) comparing Oberlin I to ICN-MCN transfer demonstrated that among patients achieving ≥ M3, patients in the ICN-MCN group took significantly longer to recover (MD = 5.25, 95% CI [2.87, 7.62]), with non-significant heterogeneity (I^2^ = 37.8%, *p* = 0.2) (Fig. 4).

**Fig. 5:**
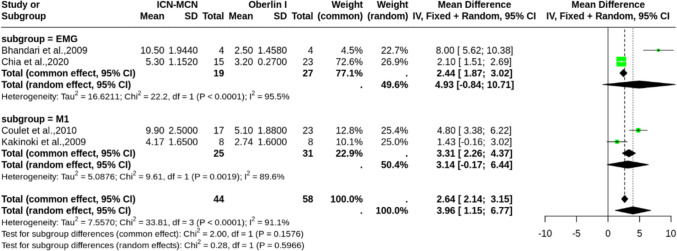
Forest plot of mean difference in time to reinnervation (Oberlin I vs. ICN-MCN), with subgroups by assessment method (EMG vs. M1)

A meta-analysis of four studies [4, 5, 7, 15] (*n* = 102) revealed a significantly faster reinnervation time in Oberlin I group (MD = 3.96, 95% CI [1.15, 6.77]), though the analysis was marked by substantial heterogeneity (I^2^ = 91.1%, *p* < 0.0001) (Fig. 5). A leave-one-out sensitivity analysis showed that omitting Coulet et al. [7] would yield insignificant results (Online Resource 8).

### Morbidity results

The reporting of complications was inconsistent, with studies frequently not reporting, denying or specifying on these outcomes, as detailed in the table **(**Online Resource 9**)**.

Pneumothorax was reported ICN-MCN patients exclusively across two studies [15, 16] with a total of four (2/8 (25%), 2/27 (7%)). It was denied or not reported across the remaining studies and other transfers (Online Resource 9).

Regarding the wrist co-flexion, while its occurrence was mentioned in six studies but not specified in numbers in five studies [7, 15, 18, 27, 30], while one study [5] reported a 100% incidence (23/23) in its Oberlin I group but a 0% rate (0/15) in its ICN-MCN group (Online Resource 9).

For donor site morbidity, a total of 18 sensory deficits were reported across three studies [4, 15, 30] distributed across groups as follows: Oberlin I (3/40 (8%); 4/4 (100%); 5/8 (63%)), Oberlin II (2/10 (20%)), and MN to MCN (4/15 (27%)) transfer groups. There were also 10 motor deficits reported in two studies [4, 30] were documented distributed across groups as follows: Oberlin I (6/40 (15%)), Oberlin II (3/10 (30%)), and MN to MCN (1/15 (7%)) groups. No donor site morbidity events were recorded for the ICN transfer groups where data was available (Online Resource 9).

Donor-site morbidities associated with fascicular transfers were often reported as transient. Sensory deficits are typically resolved within days to months [15, 30] and motor deficits, also recovered within months to around a year [4, 30]. In contrast, wrist co-flexion (secondary to the Steindler effect) appears to be a persistent neuromuscular pattern. EMG studies have confirmed its presence at long-term follow-up (mean 37 months), suggesting it is a lasting functional alteration rather than a temporary side effect [5].

## Discussion

Over recent years, surgeons have increasingly preferred fascicular nerve transfers, such as the Oberlin procedures, to restore elbow flexion [8]. As a result, these procedures have become more commonly reported in clinical studies transfers for elbow flexion compared with ICN transfers [8]. This shift is largely due to their relative technical simplicity and high reproducibility, combined with quicker results, allowing patients to achieve functional gains months earlier than with alternative procedures [15, 16]. Despite their popularity, a clear consensus on whether fascicular transfers are superior to alternatives like ICN transfers in the long term remains debatable.

Our analysis initially showed an advantage for fascicular transfers; however, this appears to be driven by the tendency of employing ICN to pan-BPI cases. When analysis was restricted to upper-BPI focused studies, it revealed that both the Oberlin I and ICN transfers yielded comparable final muscle strength, a conclusion solidified by a complete lack of statistical heterogeneity (I^2^ = 0%).

What truly distinguished the techniques in our analysis was the speed of recovery. Patients who underwent an Oberlin I transfer regained functional elbow flexion (≥ M3) approximately five months sooner than those with ICN transfers. However, this speed comes in exchange with donor-site morbidity. Studies report iatrogenic 18 sensory and 10 motor deficits in the fascicular transfer groups, this inherent risk to sacrificing fascicles from healthy UN and/or MN. Furthermore, fascicular transfers carry the risk of wrist co-flexion secondary to the Steindler effect (the phenomenon where elbow flexion is achieved through contraction of wrist and finger flexors), which we believe is underreported despite being significant. It most likely occurs due signal diversion through transferred and non-transferred fascicles and this is hypothesized to be associated with the rapid recovery in the fascicular groups, due to the synergistic action of forearm contraction aiding elbow flexion [5]. However, this on broader perspective and on the long run compromise the hand function, making it frequently undesirable [5]. These complications are less disturbing in ICN-MCN as its distribution is less vital to the upper limb function and have a better rehabilitation course on the long run, compared with UN and/or MN, where the patient synchronize elbow flexion with breathing [15]. The primary iatrogenic risk for ICN transfer is pneumothorax, which occurs if the pleura is breached during nerve harvest [17]. While this is a serious complication, its incidence can be minimized through meticulous surgical techniques, such as careful subperiosteal dissection and hydro dissection, making it a manageable, though not entirely preventable risk [17]. Also, this risk is potentially increased with later harvest for Free Functional Muscle Transfer (FFMT) when distal nerve transfer fails.

In the absence of significant functional advantage, we believe the surgical decision should consider several factors: the surgeon expertise and the patient's life and goals. While the faster recovery with fascicular transfers is strong, the risk of compromising independent hand and wrist function is a severe trade-off. For any patient who relies on fine motor skills—whether for their profession or their quality of life—we contend that the ICN transfer is the superior choice. In our opinion, accepting a slower recovery and a different risk profile is a reasonable price to pay to protect the intricate function of the hand from the permanent disruption caused by fascicular transfers.

### Previous literature

A previous meta-analysis by Vernon Lee et al. [32] is the only relevant meta-analysis that we have found whose methodology differed significantly. They included 64 studies with 1,335 adults who underwent various reconstructive procedures that aimed to restore elbow flexion. The study performed single-armed meta-analysis and had concluded Oberlin II as the procedure associated with optimal outcomes. However, we believe the study limitations reduce the certainty of this conclusion, as the single-armed pooling doesn’t account for within- and between-study differences. In contrast, the double-armed comparisons employed in our meta-analysis mitigate the impact of this bias by comparing groups from the same study directly, which helps distribute confounding factors equally among analysis arms, yielding more accurate and highly homogenous results, as present in our upper-BPI meta-analysis (I^2^ = 0%).

### Limitations

Our analysis was focused on the MRC scale as the main outcome, which is a valid outcome, however it’s operator dependent. Also, our analysis involved retrospective studies for the most part, and there was limited data on the postoperative complications of these studies. Reda et al. and Hamza et al., had conducted their studies in the same institution (Zagazig University Hospitals), which might carry some risk of cohort overlap, however each study had different time frames (2008–2011, and 2015–2018).

### Future research recommendations

We strongly recommend high-quality, prospective cohort studies with pre-defined protocols that stratify patients by injury severity to avoid the confounding that affected previous analyses. These studies should adopt a standardized core outcome set that includes not only MRC grades but also objective measures such as dynamometer-based strength assessment and serial EMG to accurately quantify recovery speed and power. Critically, a quantitative synthesis of complication rates is needed; therefore, future studies must systematically report on key adverse events, particularly donor-site morbidity and the functional impact of co-contraction, which our review found to be inconsistently documented.

## Conclusion

In patients with upper-BPIs, fascicular and ICN transfers yield comparable elbow flexion strength. The choice of procedure is a trade-off between the faster recovery offered by fascicular transfers and the better rehabilitation course of ICN transfers.

## Supplementary Information

Below is the link to the electronic supplementary material.

Supplementary Material 1 (DOCX 150 KB)

## Data Availability

All data generated or analyzed during this study are included in this published article and its supplementary information files.

## List of abbreviations

BPI: Brachial Plexus Injury
CI: Confidence Interval
EMG: Electromyography
GRADE: Grading of Recommendations, Assessment, Development, and Evaluation
ICN: Intercostal Nerve
MCN: Musculocutaneous Nerve
MD: Mean Difference
MN: Median Nerve
MRC: Medical Research Council
NOS: Newcastle-Ottawa Scale
NR: Not Reported
NS: Not Specified
PRISMA: Preferred Reporting Items for Systematic Reviews and Meta-Analyses
PROSPERO: International Prospective Register of Systematic Reviews
RR: Risk Ratio
SD: Standard Deviation
UN: Ulnar Nerve
