# Supplementary material for: Nerve transfer to musculocutaneous for elbow flexion restoration in brachial plexus injury (Ulnar and/or Median vs. Intercostal): A systematic review and meta-analysis of comparative studies
Source: Acta Neurochir (Wien). 2025 Aug 26;167(1):229. doi: 10.1007/s00701-025-06650-0 (PMC12380866; doi:10.1007/s00701-025-06650-0)
Supplement: Supplementary file 1 — Supplementary Material 1 (DOCX 150 KB) [file 701_2025_6650_MOESM1_ESM.docx]

**Nerve Transfer to Musculocutaneous for Elbow Flexion Restoration in Brachial Plexus Injury (Ulnar and/or Median vs. Intercostal): A Systematic Review and Meta-Analysis of Comparative Studies**

**Authors.** Mohamed K. A. Genedy^1^, Esraa Y. Salama^2^, Mohamed Ashraf Elsaadany^1^, Mohamed A. F. AbdelWahab^1^**,** Ahmed Fathy Amin^2^, Ahmed A. Lashin^2^, Ahmed O. Sabry, MD, MSc ^3^

**Affiliations.**

1. Faculty of Medicine, Cairo University, Cairo, Egypt
2. Faculty of Medicine, Benha University, Benha, Egypt
3. Orthopedic Surgery Department, Cairo University, Cairo, Egypt.

* **Corresponding author:** Ahmed O. Sabry

- **Affiliation:** Orthopedic Surgery Department, Cairo University, Cairo, Egypt.
- **Postal address:** El Saray Street Manial - El Manial, Cairo, Egypt : 11956
- **Telephone number:** (+20) 1203399233
- **E-mail:** [ahmed.o.sabry@kasralainy.edu.eg](mailto:ahmed.o.sabry@kasralainy.edu.eg)

Mohamed K. A. Genedy

Email: mo7med.kamel2017@gmail.com

ORCID: 0009-0009-8234-1557

Affiliation: Faculty of Medicine, Cairo University, Cairo, Egypt

Esraa Y. Salama

Email: esraa.yasser.salama251@gmail.com

ORCID: 0000-0003-4640-4513

Affiliation: Faculty of Medicine, Benha University, Qalyubia, Egypt

Mohamed Ashraf Elsaadany

Email: moashrafalsaadany@gmail.com

ORCID: 0009-0005-2050-215X

Affiliation: Faculty of Medicine, Cairo University, Cairo, Egypt

Mohamed A. F. AbdelWahab

Email: mohamedali52035@gmail.com

ORCID: 0009-0009-5517-8205

Affiliation: Faculty of Medicine, Cairo University, Cairo, Egypt

Ahmed Fathy Amin

Email: ahmedfathyamin427@gmail.com

ORCID: 0009-0005-0433-8134

Affiliation: Faculty of Medicine, Benha University, Qalyubia, Egypt.

Ahmed A. Lashin

Email: ahmed180511@fmed.bu.edu.eg

ORCID: 0009-0000-9826-6664

Affiliation: Faculty of Medicine, Benha University, Qalyubia, Egypt

Ahmed O. Sabry

Email: ahmed.o.sabry@kasralainy.edu.eg

ORCID: 0000-0002-7705-0335

Affiliation: Orthopedic Surgery Department, Cairo University, Cairo, Egypt.

**Contents**

**Online Resource 1** Search strategy table.

**Online Resource 2** Summary of the Included Studies Characteristics table.

**Online Resource 3** Summary of Population Characteristics table.

**Online Resource 4** Summary of Quality assessment table.

**Online Resource 5** GRADE assessment for the outcome achieving ≥M3.

**Online Resource 6** Funnel plot for the meta-analysis of achieving ≥M3 recovery (Oberlin I vs. ICN-MCN) in the overall cohort figure. Each point represents an individual study.

**Online Resource 7** Leave-one-out analysis of the risk ratio for achieving ≥M3 motor recovery (Oberlin II vs. ICN-MCN) in the overall cohort figure**.**

**Online Resource 8** Leave-one-out analysis of the mean difference in time to reactivation (Oberlin I vs. ICN-MCN) in the upper-BPI cohort figure**.**

**Online Resource 9** Summary of the Morbidities table.

| **Data Base** | **Search Strategy** | **Number Of Results** |
| --- | --- | --- |
| **PubMed** | (nerve* OR root* OR fascicle* OR fascicular*) AND (transfer* OR neurotization* OR neurotisation* OR translocation* OR "cross-over" OR crossover OR transport* OR procedure* OR technique*) AND (median OR ulnar OR oberlin) AND (intercostal) | All Fields = 254  Results |
| **Embase** | ((nerve* or root* or fascicle* or fascicular*).ti,ab.) AND ((transfer* or neurotization* or neurotisation* or translocation* or "cross-over" or crossover or transport* or procedure* or technique*).ti,ab.) AND ((median or ulnar or oberlin).ti,ab.) AND (intercostal or intercostal nerve).ti,ab.) | Basic search = 739 Results |
| **Scopus** | ( nerve* OR root* OR fascicle* OR fascicular* ) AND ( transfer* OR neurotization* OR neurotisation* OR translocation* OR "cross-over" OR crossover OR transport* OR procedure* OR technique* ) AND ( median OR ulnar OR oberlin ) AND ( intercostal ) | Abstract, Title, Author = 399 Results |
| **Web Of Science (Core Collection)** | (nerve* or root* OR fasicle* or fasicular*) AND (transfer* OR neurotization* OR neurotisation* OR translocation* OR “cross?over” OR transport* OR procedure* OR technique*) AND (median OR ulnar OR oberlin) AND (intercostal) | All Fields = 262 Results |

***Online Resource 1*** *Search strategy table.*

| Study Name, Year | Intervention Groups | Country | Study Design | Sample Size (N) | Time to surgery  (mean/SD in months) | Follow-up period   (mean/SD in months) | Fascicles/ICNs Transferred (N) |
| --- | --- | --- | --- | --- | --- | --- | --- |
| Songcharoen et al., 2005 | Oberlin I | Thailand | R | 40 | NR | 24 | 1 |
|  | MN-MCN |  |  | 15 | NR | 24 | 1 |
|  | ICN-MCN |  |  | 22 | NR | 48 | 2 |
| Bhandari et al., 2009 | Oberlin I | India | R | 4 | 4.2 ± 1.23 | 23.6 ± 3.5 | ≥1 |
|  | Oberlin II |  |  | 10 | 3.75 ± 0.5 | 23.6 ± 3.5 | ≥2 |
|  | ICN-MCN |  |  | 4 | 3.5 ± 0.58 | 23.75 ± 8.9 | 3 |
| Chia et al., 2020 | Oberlin I | Japan | R | 23 | 4 ± 1.07 | 37 ± 41.54 | ≥1 |
|  | ICN-MCN |  |  | 15 | 4 ± 0.86 | 58 ± 64.512 | 3 |
| Coulet et al., 2010 | Oberlin I | France and Argentina | R | 23 | 6.5 ± 2.8 | 25 ± 13.6 | 2 |
|  | ICN-MCN |  |  | 17 | 5.7 ± 2.3 | 32 ± 21.9 | 3 |
| Emamhadi et al., 2021 | Oberlin I | Iran | R | 24 | NR | 13.3 ± NR | 1 |
|  | ICN-MCN |  |  | 15 | NR | 23.3 ± NR | 3 |
| Hamza et al., 2019 | Oberlin I | Egypt | R | 4 | 6 ± 1.41 | 19.75 ± 12.01 | ≥1 |
|  | Oberlin II |  |  | 5 | 5.4 ± 1.34 | 15.6 ± 3.58 | ≥2 |
|  | ICN-MCN |  |  | 12 | 6.75 ± 2 | 20.08 ± 8.47 | 3 |
| Kakinoki et al., 2010 | Oberlin I | Japan | R | 8 | 4.66 ± 0.78 | 32.36 ± 26.62 | 2 |
|  | ICN-MCN |  |  | 8 | 4.49 ± 1.12 | 30.06 ± 11.55 | 2 |
| Kang et al., 2020 (overall) | Oberlin I | Singapore | R | 8 | 3.81 ± 1.07 | ≥ 24 | 1 |
|  | MN-MCN |  |  | 3 | 1.5 ± 1.32 |  | 1 |
|  | ICN-MCN |  |  | 27 | 3.67 ± 1.57 |  | 3 |
| Kang et al., 2020  (upper BPI) | Oberlin I | Singapore | R | 8 | 3.81 ± 1.07 | ≥ 24 | 1 |
|  | MN-MCN |  |  | 3 | 1.5 ± 1.32 |  | 1 |
|  | ICN-MCN |  |  | 10 | 3.55 ± 1.62 |  | 3 |
| Lee et al., 2023 (1st decade) | Oberlin I | Taiwan | R | 34 | NR | NR | NR |
|  | ICN-MCN |  |  | 29 |  |  | NR |
| Lee et al., 2023(2nd decade) | Oberlin II | Taiwan | R | 68 | NR | NR | NR |
|  | ICN-MCN |  |  | 5 |  |  | NR |
| Mandal et al., 2018 | Oberlin II | India | P | 20 | 6.7 ± 3.16 | 18 ± 0 | 2 |
|  | ICN-MCN |  |  | 20 |  |  | 3 |
| Reda et al., 2012 | Oberlin I | Egypt | R | 6 | 6.32 ± 4.22 | 18.88 ± 8.22 | 1 |
|  | ICN-MCN |  |  | 8 |  |  | 3 |
| Vancea et al.,2025 | Oberlin II | Romania | R | 11 | NR | NR | 2 |
|  | ICN-MCN |  |  | 13 |  |  | 3 |
| Romeih and Mazrou, 2025 | Oberlin II | Egypt | P | 19 | 2.89 ± 1.7 | ≥ 60 | 2 |
|  | ICN-MCN |  |  | 17 | 3.65 ± 1.5 |  | 3 |

***Online Resource 2*** *Summary of the Included Studies Characteristics table.*

| Study Name, Year | Intervention Groups | Upper BPI (N) | Upper Extended BPI (N) | Total BPI (N) | Age in years (Mean ± SD) | Gender (M: F) |
| --- | --- | --- | --- | --- | --- | --- |
| Songcharoen et al., 2005 | Oberlin I | 40 | 0 | 0 | NR | NR |
|  | MN-MCN | 15 | 0 | 0 |  |  |
|  | ICN-MCN | 0 | | 22 |  |  |
| Bhandari et al., 2009 | Oberlin I | 4 | 0 | 0 | 24.5 ± 5.07 | 4:0 |
|  | Oberlin II | 8 | 2 | 0 | 27.3 ± 6.5 | 10:0 |
|  | ICN-MCN | 0 | 4 | 0 | 22.5 ± 2.52 | 4:0 |
| Chia et al., 2020 | Oberlin I | 11 | 12 | 0 | 32 ± 10.99 | 22:1 |
|  | ICN-MCN | 2 | 13 | 0 | 33 ± 10.66 | 14:1 |
| Coulet et al., 2010 | Oberlin I | 18 | 5 | 0 | 28 ± 10.2 | 20:3 |
|  | ICN-MCN | 4 | 13 | 0 | 25 ± 8.5 | 15:2 |
| Emamhadi et al., 2021 | Oberlin I | 24 | | 0 | 31.7 ± 11.1 | 21:3 |
|  | ICN-MCN | 0 | | 15 | 31.3 ± 10.4 | 11:4 |
| Hamza et al., 2019 | Oberlin I | 4 | | 0 | 40 ± 16.65 | 3:1 |
|  | Oberlin II | 5 | | 0 | 25.4 ± 8.23 | 4:1 |
|  | ICN-MCN | 0 | | 12 | 27.67 ± 13.55 | 12:0 |
| Kakinoki et al., 2010 | Oberlin I | 7 | 1 | 0 | 37.88 ± 15.94 | 6:2 |
|  | ICN-MCN | 3 | 5 | 0 | 38.38 ± 17.6 | 7:1 |
| Kang et al., 2020 (overall) | Oberlin I | 2 | 6 | 0 | 32.5 ± 15.44 | 10:1 |
|  | MN-MCN | 1 | 2 | 0 | 28 ± 12.29 |  |
|  | ICN-MCN | 1 | 9 | 17 | 24.67 ± 9.39 | 25:2 |
| Kang et al., 2020  (upper BPI) | Oberlin I | 2 | 6 | 0 | 32.5 ± 15.44 | 10:1 |
|  | MN-MCN | 1 | 2 | 0 | 28 ± 12.29 |  |
|  | ICN-MCN | 1 | 9 | 0 | 23.1 ± 4.89 | NR |
| Lee et al., 2023  (1st decade) | Oberlin I | 34 | | 0 | NR | NR |
|  | ICN-MCN | 29 | | 0 |  |  |
| Lee et al., 2023  (2nd decade) | Oberlin II | 68 | | 0 | NR | NR |
|  | ICN-MCN | 5 | | 0 |  |  |
| Mandal et al., 2018 | Oberlin II | 12 | 8 | 0 | 31.78 ± 6.7 | 39:1 |
|  | ICN-MCN | 0 | | 20 |  |  |
| Reda et al., 2012 | Oberlin I | 4 | 2 | 0 | NR | NR |
|  | ICN-MCN | 0 | | 8 |  |  |
| Vancea et al.,2025 | Oberlin II | 11 | | 0 | NR | NR |
|  | ICN-MCN | 0 | | 13 |  |  |
| Romeih and Mazrou, 2025 | Oberlin II | 10 | 9 | 0 | 31.68 ± 15.44 | 17:2 |
|  | ICN-MCN | 4 | 13 | 0 | 28.41 ± 10.79 | 12:5 |

***Online Resource 3*** *Summary of Population Characteristics table.*

| Study ID | **Selection** | | | **Comparability** | | | **Outcome** | | | **Overall Score** | |
| --- | --- | --- | --- | --- | --- | --- | --- | --- | --- | --- | --- |
|  | 1 | 2 | 3 | 4 | 5 | 6 | 7 | 8 | 9 | Total number of stars | Quality |
| Songcharoen et al. 2005 | * | * | * | * |  |  |  | * | * | 6 out of 9 | Moderate |
| Bhandari et al. 2009 | * | * | * | * |  |  |  | * | * | 6 out of 9 | Moderate |
| Coulet et al. 2010 |  | * | * | * |  |  | * | * | * | 6 out of 9 | Moderate |
| Kakinoki et al. 2010 | * | * | * | * |  |  | * | * | * | 7 out of 9 | Good |
| Mohammad-Reda A. 2012 | * | * | * | * |  |  |  | * | * | 6 out of 9 | Moderate |
| Mandal et al. 2018 | * |  | * | * |  |  |  | * | * | 5 out of 9 | Moderate |
| Hamza et al. 2019 | * |  | * | * |  |  |  |  |  | 3 out of 9 | Poor |
| Chia et al. 2020 |  | * | * | * | * |  | * | * | * | 7 out of 9 | Good |
| Kang et al. 2020 | * | * | * | * | * | * |  | * |  | 7 out of 9 | Good |
| Emamhadi et al. 2021 |  |  | * | * |  |  |  | * |  | 3 out of 9 | Poor |
| Lee et al. 2023 | * | * | * | * |  |  |  | * | * | 6 out of 9 | Moderate |
| Romeih & Mazrou 2025 | * | * | * | * | * |  |  | * | * | 7 out of 9 | Good |
| Vancea et al. 2025 | * | * | * | * |  |  |  | * | * | 6 out of 9 | Moderate |

***Online Resource 4*** *Summary of Quality assessment table.*

| **Certainty assessment** | | | | | | | **№ of patients** | | **Effect** | | **Certainty** | **Importance** |
| --- | --- | --- | --- | --- | --- | --- | --- | --- | --- | --- | --- | --- |
| **№ of studies** | **Study design** | **Risk of bias** | **Inconsistency** | **Indirectness** | **Imprecision** | **Other considerations** | **[intervention]** | **[comparison]** | **Relative  (95% CI)** | **Absolute  (95% CI)** |  |  |
| **Achieving M3 or more in overall studies (Oberlin I vs ICN-MCN)** | | | | | | | | | | | | |
| 10 | non-randomised studies cohort | serious | not serious | not serious | not serious | none | 115/157 (73.2%) | 155/174 (89.1%) | RR 0.84  (0.75 to 0.94) | 143 fewer per 1,000  (from 223 fewer to 53 fewer) | ⨁◯◯◯  Very low | Critical |

***Online Resource 5*** *GRADE assessment for the outcome achieving ≥M3.*


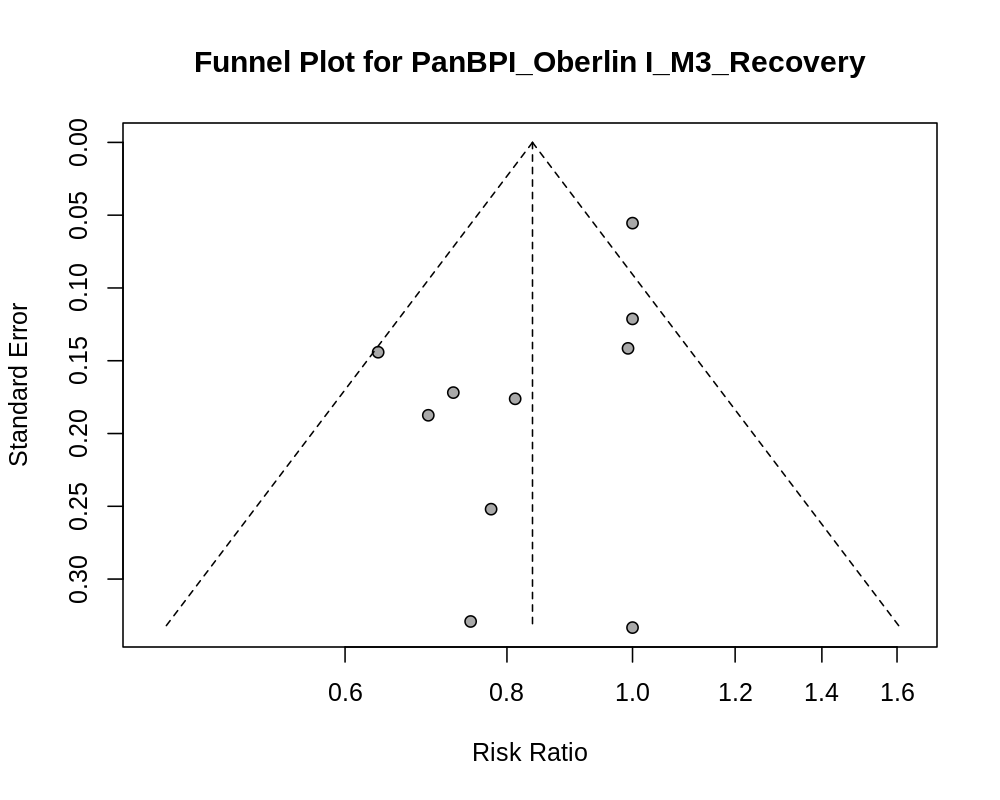


***Online Resource 6*** *Funnel plot for the meta-analysis of achieving ≥M3 recovery (Oberlin I vs. ICN-MCN) in the overall cohort. Each point represents an individual study.*


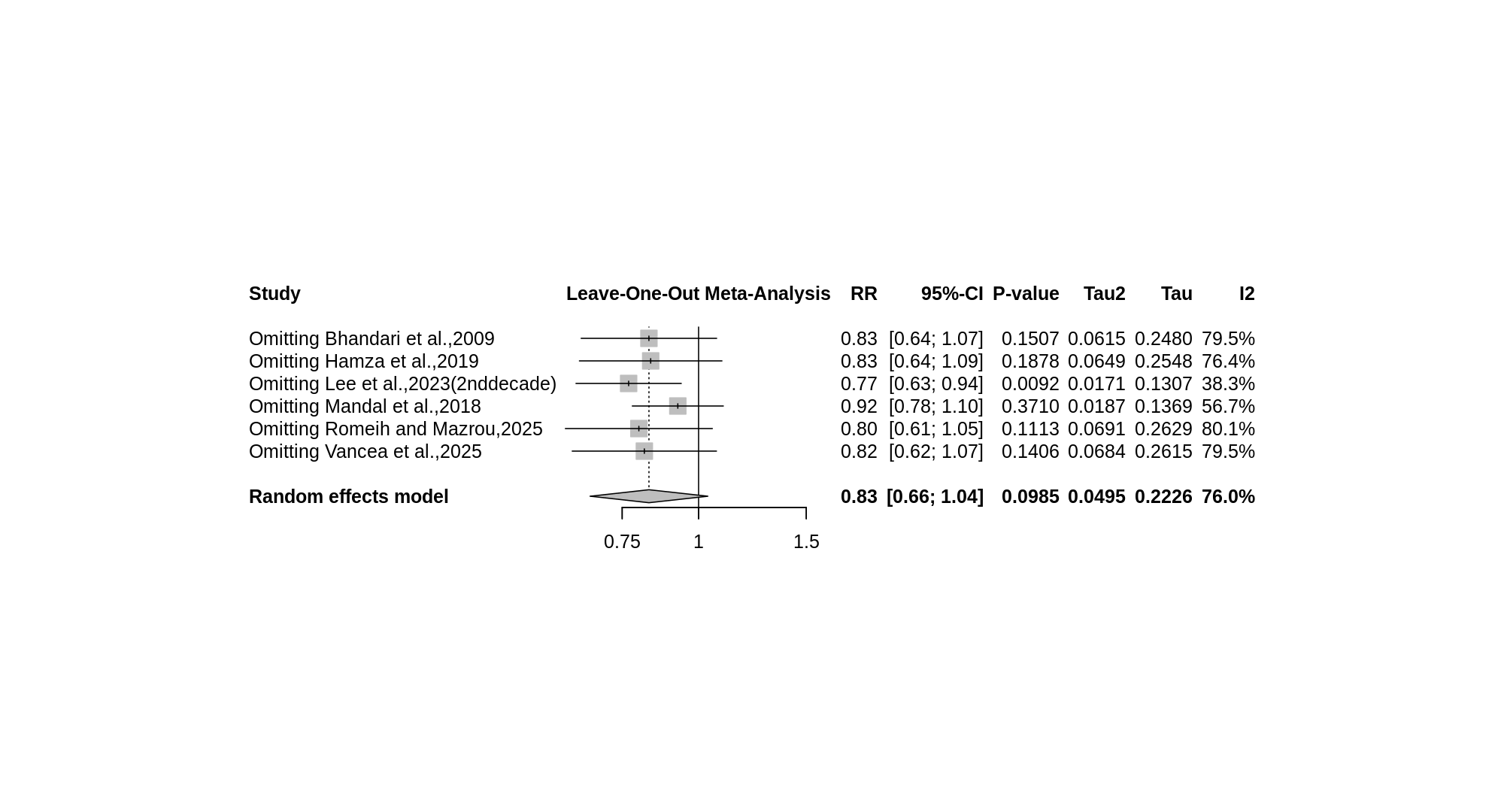


***Online Resource 7*** *Leave-one-out analysis plot of the risk ratio for achieving ≥M3 motor recovery (Oberlin II vs. ICN-MCN) in the overall cohort.*


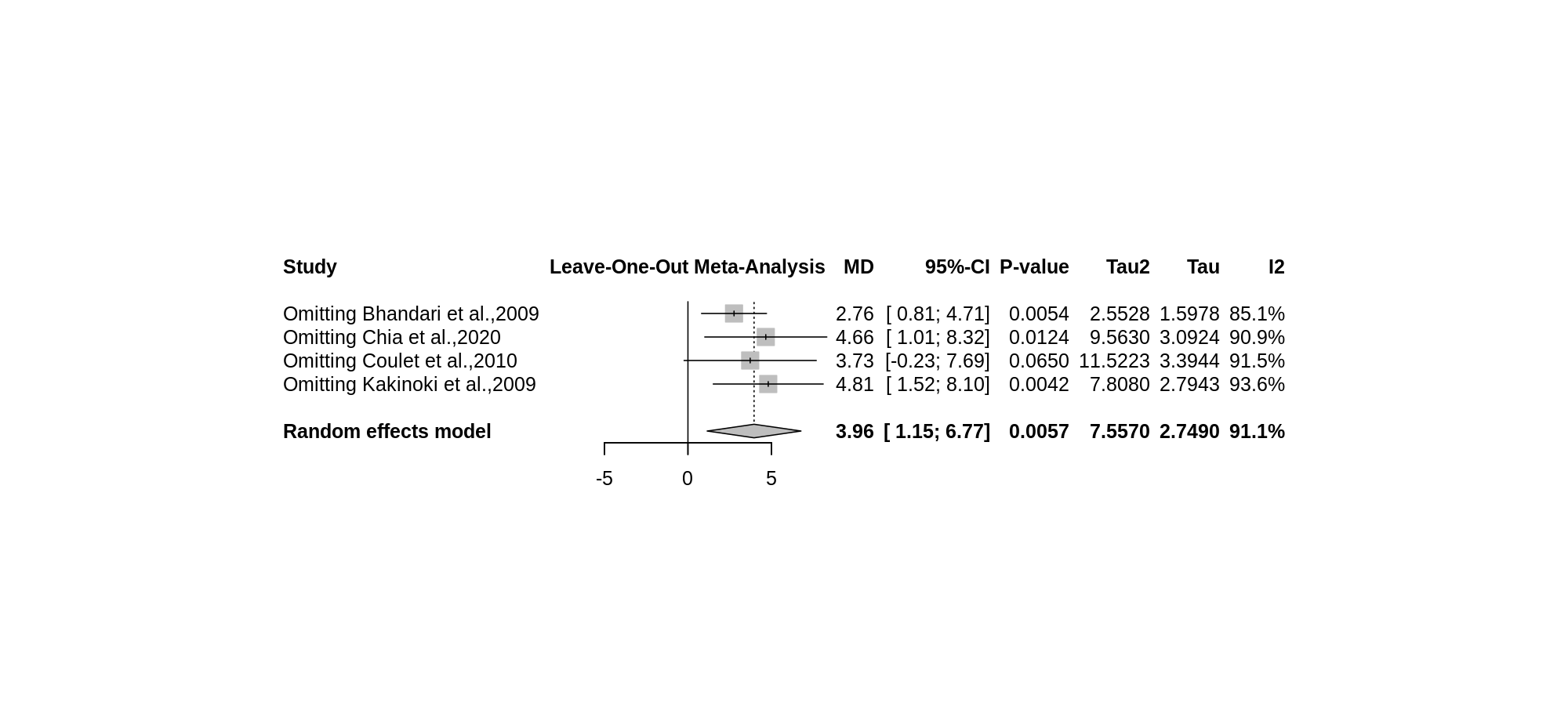


***Online Resource 8*** *Leave-one-out analysis plot of the mean difference in time to reactivation (Oberlin I vs. ICN-MCN) in the upper-BPI cohort.*

| Study Name, Year | Complication | Oberlin I (event/total (%)) | Oberlin II (event/total (%)) | ICN-MCN (event/total (%)) | MN-MCN (event/total (%)) |
| --- | --- | --- | --- | --- | --- |
| Songcharoen et al., 2005 | Pneumothorax | NS | — | NS | — |
|  | Wrist Co-flexion | NR | — | NS | — |
|  | Donor Sensory Morbidity | 3/40 (8%) | — | NS | 4/15 (27%) |
|  | Donor Motor Morbidity | 6/40 (15%) | — | 0/22 (0%) | 1/15 (7%) |
| Bhandari et al., 2009 | Pneumothorax | 0/4 (0%) | 0/10 (0%) | 0/4 (0%) | — |
|  | Wrist Co-flexion | NR | — | — | — |
|  | Donor Sensory Morbidity | 4/4 (100%) | 2/10 (20%) | 0/4 (0%) | — |
|  | Donor Motor Morbidity | 0/4 (0%) | 3/10 (30%) | 0/4 (0%) | — |
| Chia et al., 2020 | Pneumothorax | 0/23 (0%) | — | 0/15 (0%) | — |
|  | Wrist Co-flexion | 23/23 (100%) | — | 0/15 (0%) | — |
|  | Donor Sensory Morbidity | 0/23 (0%) | — | 0/15 (0%) | — |
|  | Donor Motor Morbidity | 0/23 (0%) | — | 0/15 (0%) | — |
| Coulet et al., 2010 | Pneumothorax | 0/23 (0%) | — | 0/17 (0%) | — |
|  | Wrist Co-flexion | NS | — | — | — |
|  | Donor Sensory Morbidity | 0/23 (0%) | — | 0/17 (0%) | — |
|  | Donor Motor Morbidity | 0/23 (0%) | — | 0/17 (0%) | — |
| Emamhadi et al., 2021 | Pneumothorax | NR | — | — | — |
|  | Wrist Co-flexion | NR | — | — | — |
|  | Donor Sensory Morbidity | 0/24 (0%) | — | 0/15 (0%) | — |
|  | Donor Motor Morbidity | 0/24 (0%) | — | 0/15 (0%) | — |
| Hamza et al., 2019 | Pneumothorax | NR | NR | NR | — |
|  | Wrist Co-flexion | NR | NR | NR | — |
|  | Donor Sensory Morbidity | NS | NR | — | — |
|  | Donor Motor Morbidity | NS | NR | — | — |
| Kakinoki et al., 2010 | Pneumothorax | 0/8 (0%) | — | 2/8 (25%) | — |
|  | Wrist Co-flexion | NS | — | — | — |
|  | Donor Sensory Morbidity | 5/8 (63%) | — | 0/8 (0%) | — |
|  | Donor Motor Morbidity | 0/8 (0%) | — | 0/8 (0%) | — |
| Kang et al., 2020 (overall) | Pneumothorax | 0/8 (0%) | — | 2/27 (7%) | 0/3 (0%) |
|  | Wrist Co-flexion | NR | — | — | — |
|  | Donor Sensory Morbidity | 0/8 (0%) | — | 0/27 (0%) | 0/3 (0%) |
|  | Donor Motor Morbidity | 0/8 (0%) | — | 0/27 (0%) | 0/3 (0%) |
| Kang et al., 2020 (upper BPI) | Pneumothorax | 0/8 (0%) | — | 1/10 (10%) | 0/3 (0%) |
|  | Wrist Co-flexion | NR | — | — | — |
|  | Donor Sensory Morbidity | 0/8 (0%) | — | 0/10 (0%) | 0/3 (0%) |
|  | Donor Motor Morbidity | 0/8 (0%) | — | 0/10 (0%) | 0/3 (0%) |
| Lee et al., 2023 (1st decade) | Pneumothorax | NR | — | — | — |
|  | Wrist Co-flexion | NS | — | — | — |
|  | Donor Sensory Morbidity | NR | — | — | — |
|  | Donor Motor Morbidity | NR | — | — | — |
| Lee et al., 2023 (2nd decade) | Pneumothorax | — | NR | — | — |
|  | Wrist Co-flexion | — | NS | — | — |
|  | Donor Sensory Morbidity | — | NR | — | — |
|  | Donor Motor Morbidity | — | NR | — | — |
| Mandal et al., 2018 | Pneumothorax | — | 0/20 (0%) | 0/20 (0%) | — |
|  | Wrist Co-flexion | — | NR | NR | — |
|  | Donor Sensory Morbidity | — | NS | — | — |
|  | Donor Motor Morbidity | — | NS | — | — |
| Reda et al., 2012 | Pneumothorax | NR | — | — | — |
|  | Wrist Co-flexion | NR | — | — | — |
|  | Donor Sensory Morbidity | NR | — | — | — |
|  | Donor Motor Morbidity | NR | — | — | — |
| Vancea et al., 2025 | Pneumothorax | — | NR | NR | — |
|  | Wrist Co-flexion | — | NR | NR | — |
|  | Donor Sensory Morbidity | — | NR | NR | — |
|  | Donor Motor Morbidity | — | NR | NR | — |
| Romeih and Mazrou, 2025 | Pneumothorax | — | NR | — | — |
|  | Wrist Co-flexion | — | NS | — | — |
|  | Donor Sensory Morbidity | — | NR | — | — |
|  | Donor Motor Morbidity | — | NR | — | — |

***Online Resource 9*** *Summary of the Morbidities Table.*

*NR: Complication Not Reportd*

*NS: Complication Not Specified*

*Zero: Complication Denied*
